# A Stacked Long Short-Term Memory Approach for Predictive Blood Glucose Monitoring in Women with Gestational Diabetes Mellitus

**DOI:** 10.3390/s23187990

**Published:** 2023-09-20

**Authors:** Huiqi Y. Lu, Ping Lu, Jane E. Hirst, Lucy Mackillop, David A. Clifton

**Affiliations:** 1 Department of Engineering Science, University of Oxford, Oxford OX3 7DQ, UK; ping.lu@eng.ox.ac.uk (P.L.); david.clifton@eng.ox.ac.uk (D.A.C.); 2 Women’s Centre, Oxford University Hospitals NHS Foundation Trust, Oxford OX3 9DU, UK; jhirst@georgeinstitute.org.uk (J.E.H.); lucy.mackillop@ouh.nhs.uk (L.M.); 3 George Institute for Global Health, Imperial College London, London W12 7RZ, UK; 4 Oxford Suzhou Centre for Advanced Research, Suzhou 215123, China

**Keywords:** patient monitoring, gestational diabetes, clinical machine learning, pregnancy care, medical informatics

## Abstract

Gestational diabetes mellitus (GDM) is a subtype of diabetes that develops during pregnancy. Managing blood glucose (BG) within the healthy physiological range can reduce clinical complications for women with gestational diabetes. The objectives of this study are to (1) develop benchmark glucose prediction models with long short-term memory (LSTM) recurrent neural network models using time-series data collected from the GDm-Health platform, (2) compare the prediction accuracy with published results, and (3) suggest an optimized clinical review schedule with the potential to reduce the overall number of blood tests for mothers with stable and within-range glucose measurements. A total of 190,396 BG readings from 1110 patients were used for model development, validation and testing under three different prediction schemes: 7 days of BG readings to predict the next 7 or 14 days and 14 days to predict 14 days. Our results show that the optimized BG schedule based on a 7-day observational window to predict the BG of the next 14 days achieved the accuracies of the root mean square error (RMSE) = 0.958 ± 0.007, 0.876 ± 0.003, 0.898 ± 0.003, 0.622 ± 0.003, 0.814 ± 0.009 and 0.845 ± 0.005 for the after-breakfast, after-lunch, after-dinner, before-breakfast, before-lunch and before-dinner predictions, respectively. This is the first machine learning study that suggested an optimized blood glucose monitoring frequency, which is 7 days to monitor the next 14 days based on the accuracy of blood glucose prediction. Moreover, the accuracy of our proposed model based on the fingerstick blood glucose test is on par with the prediction accuracies compared with the benchmark performance of one-hour prediction models using continuous glucose monitoring (CGM) readings. In conclusion, the stacked LSTM model is a promising approach for capturing the patterns in time-series data, resulting in accurate predictions of BG levels. Using a deep learning model with routine fingerstick glucose collection is a promising, predictable and low-cost solution for BG monitoring for women with gestational diabetes.

## 1. Introduction

Gestational diabetes mellitus (GDM) is one of the most common medical complications of pregnancy and is defined as carbohydrate intolerance resulting in hyperglycaemia of variable severity with onset or first recognition during pregnancy by the World Health Organization (WHO) [1]. GDM is associated with short- and long-term adverse health consequences for mothers and children. Due to increasing obesity and maternal age, the worldwide prevalence of GDM has doubled in the past five years. GDM currently affects approximately one in six pregnancies worldwide and one in five pregnancies in the UK [2,3,4,5,6]. Cost-effective, safe and clinically plausible management solutions are urgently needed to improve the quality of health and care in response to the increased demand for maternity and diabetes clinical services.

People with GDM need to self-manage their blood glucose through dietary and exercise adjustments, as well as with medication intervention. In many places around the world, including the UK, GDM management initially follows a ‘one-size-fits-all approach’ with little consideration of individual differences. Management begins with education, dietary and lifestyle advice. Capillary blood glucose (BG) testing is recommended, fasting, pre- and post-meal between 4 and 6 times a day, until the baby’s birth. Depending on the BG response, some women with GDM need oral medications or insulin intervention for their BG management, whereas some can manage BG via diet and exercise. Conscious glucose monitoring (CGM) is a more expensive alternative for glucose monitoring and management. CGM repeats the sampling of interstitial glucose by a fine capillary via an adhesive sensor on the skin. Whilst transformative for pregnant women with type 1 diabetes, CGM technologies are expensive with a lack of evidence to support their use in GDM. As such, fingerstick monitoring remains the recommended method of glucose monitoring in GDM.

Despite the need for digital health innovations in helping blood glucose management, a very limited number of clinical studies have been commercialized and put into clinical practice for blood glucose management. Instead, the majority of work is nonclinical applications for generic use. The established technologies include GDm-Health (UK), Pregnant+ (Norway, China and Nepal) and MobiGuide (Spain). For more information, Lu et al.’s paper [7] provides a comprehensive review of sensor technologies and machine learning methods in blood glucose monitoring and management. Previous work in predicting blood glucose during GDM includes medication prediction [8] and high blood glucose alert systems [9]. 

This study builds on the GDm-Health development at the Institute of Biomedical Engineering and the Nuffield Department of Women’s Reproductive Health at the University of Oxford. GDm-Health is the UK’s first clinical gestational diabetes management platform [10]. GDm-Health is a smartphone-based, Bluetooth-controlled BG monitoring platform with a bidirectional communication system between patients and clinicians to enable remote patient monitoring [11,12]. The GDm-Health device is a CE-marked medical device and can be prescribed via health providers. The GDm-Health platform is currently being used by more than 50 NHS Foundation Trust Hospitals and covers more than 50% of the gestational diabetes population in England. 

## 2. Results 

### 2.1. Study Design and Participants

Model development was performed on data from a single site at the Women’s Centre in the John Radcliffe Hospital, Oxford University (OUH) NHS Foundation Trust, Oxford, UK. Data were extracted from the electronic medical records of each woman from their first pregnancy booking appointment until the closure of that maternity encounter, following either delivery or discharge from postnatal care, loss to follow-up (e.g., moved away) or miscarriage/termination of pregnancy. 

There were 1282 patients with GDM who were managed by the OUH and used the GDm-Health Apps between 14/08/2017 and 10/05/2021. Among them, 1110 patients (1148 pregnancies) were included in this study. The mean maternal age, BMI, parity and gravidity were 33.5 years, 31.0, 1.6 and 2.6, respectively.

Eligibility criteria were pregnant women with GDM at the OUH, with singleton pregnancies, who recorded their BG data on the GDm-Health system and had an electronic health record for the pregnancy. For this study, GDM was defined as per the hospital guidelines at the time of pregnancy. Throughout the study period, all pregnant women underwent screening using clinical risk factors suggested by the UK National Institute for Health and Care Excellence (NICE). Those with risk factors underwent a 2 h 75 g glucose tolerance test (GTT). Until April 2020, GDM was diagnosed using the thresholds from WHO/IADPSG (≥5.1 fasting, ≥10.0 at 1 h, ≥8.5 mmol/L at 2 h) at approximately 24–26 weeks of pregnancy. From April 2020 to March 2021, GDm diagnosis was based on the RCOG COVID pathway (fasting glucose ≥ 5.3 mmol/L, HbA1c ≥ 39 mmol/mol at approximately 28 weeks). Following March 2021, NICE thresholds for the GTT were used to diagnose GDM (5.3 fasting, 7.8 mmol/L 2 h). We excluded women who had not consented for their anonymised data to be shared through GDm-Health and women who opted out of the use of their data in health research.

**Figure 1:**
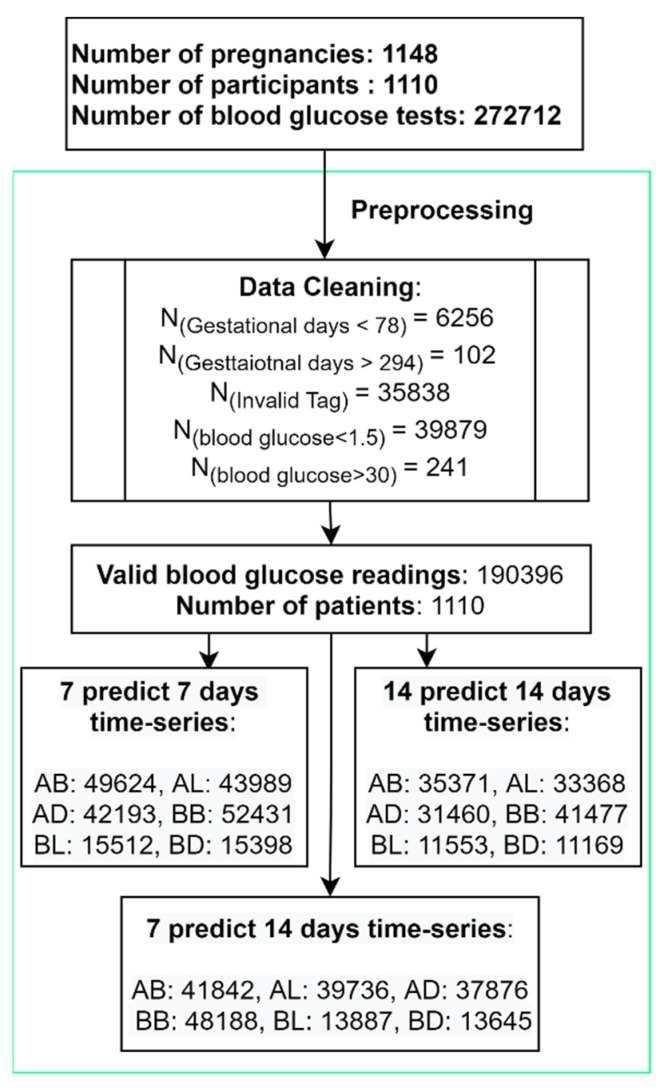
Flowchart of participants, data cleaning and model preparation.

Women using the GDm-Health system to monitor their BG levels were advised to test a minimum of three days of the week, measuring their BG four times a day (before breakfast (BB), one hour after breakfast (AB), one hour after lunch (AL) and one hour before dinner (BD)). Before-lunch (BL) and before-dinner (BD) tests were also recommended when taking glucose-lowering medication (metformin or insulin). We refer to these six measurements as “Tags AB, AL, AD, BB, BL, BD” in this paper. The flowchart of patients, data preprocessing and the number of time series used in the three models are reported in Figure 1.

**Figure 2:**
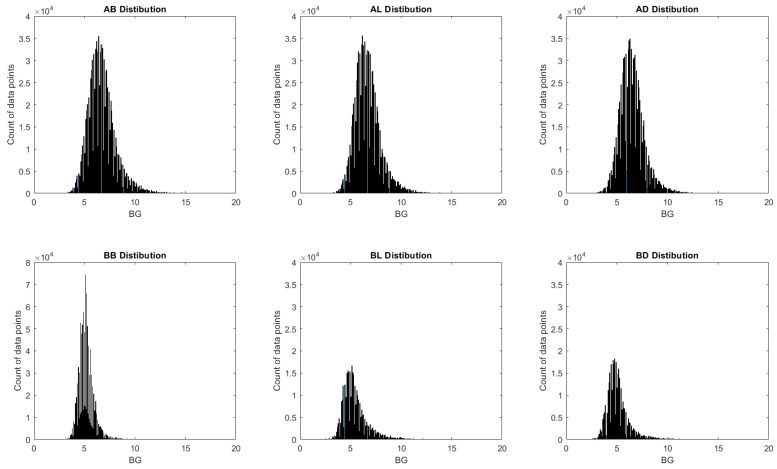
Distribution of BG readings for 7-days-predict-14-days (in the order of AB, AL, AD, BB, BL, BD left to right and then the first to the second row), as an exemplar.

The distributions of BG readings after window preparations are shown in Figure 2. In general, all six tags demonstrated a good fit of normal distribution with one peak and moderate symmetricity. The distribution of all after-meal measurements demonstrated good symmetrical distributions, and all before-meal measurements had a small tail (stew right) that represented the Hyper group. This observation matches the time series size reported in Table 1.

Beyond the time-series BG readings of three prediction windows, readings were subgrouped into Hyper, Normal and Hypo, which refers to the value of glucose measurements above, within or under the NICE guidance [13]. The Hyper (High) group had one or more BG readings >7.8 mmol/L for 1 h postprandial measurements or >5.3 mmol/L for fasting measurements within the observation window. The Normal group had all readings within the target range, and the Hypo group (Low) had at least one reading <3.0 in the observation window.

### 2.2. Model Performance

**Figure 3:**
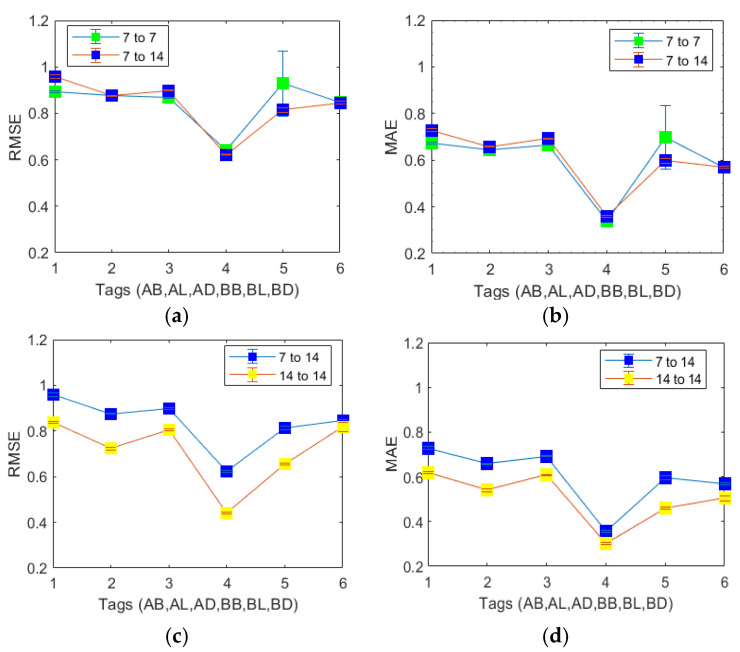
LSTM prediction performance comparisons: (**a**) RMSE results of 7-days-predict-7-days vs. 7-days-predict-14 days, (**b**) MAE results of 7-days-predict-7-days vs. 7-days-predict-14-days, (**c**) RMSE results of 7-days-predict-14-days vs. 14-days-predict-14-days and (**d**) MAE results of 7-days-predict-14-days vs. 14-days-predict-14-days.

We first developed and tested the Stacked LSTM models for 7-days-predict-7-days, 7-days-predict-14-days and 14-days-predict-14 days for all windows. By comparing the three models’ mean absolute error (MAE) and root mean square error (RMSE) side by side, as shown in Figure 3, we can confirm that (1) before-breakfast is the most “predictable” BG measurement among all six before- and post-meal measurements and (2) the 7-days-predict-14 days is the optimized BG monitoring timeframe.

We then chose the 7-day-predicted 14-day window frame as an example to evaluate the performance of the Stacked LSTM model under two subgroups, namely, the Normal and Hyper subgroups, as defined in the Methods section. Our system provides an alert for women who have hypoglycaemia no prediction model was trained on the Hypo subgroup data. The characteristics and model performance of the subgroups are listed in Table 1. The results shown in Table 1 confirmed that the BG prediction in the Normal subgroup had smaller RMSE and MAE values than those in the Hyper subgroup. In conclusion, all pre-meal predictions are more accurately predictable than the post-meal predictions. For the Overall group (shown in Figure 3) and the Normal and Hyper subgroups, the RMSE values are higher than the MAE values, suggesting a moderate number of large errors during model testing.

In Table 2, we summarize the study design and the performance of the current state-of-the-art BG prediction models using the CGM data, and then compare them with our model. The comparison results confirmed that the stacked LSTM deep learning model can achieve excellent glucose prediction performance similar to that of the CGM system. Fingerstick BG tests can be seen as a snapshot of CGM. The “event” labels AB, AL, AD, BB, BL and BD naturally captured the oscillation of the BG pattern. The compression results demonstrated that by using routine glucose collection schedules (4–6 times a day) under clinical platforms such as GDm-Health or similar fingerstick glucose management systems, we can use the stacked LSTM model as a promising approach to capture the patterns and seasonality in BG data.

## 3. Conclusions and Discussion

Innovations derived from digital health and machine learning have the potential to improve clinical care delivery and personalize glucose monitoring schedules for people with GDM [18]. The James Lind Alliance Priority Setting Partnerships suggested that a top research priority for women’s health during pregnancy was to use digital health technology to improve pregnancy, birth and mother and child health outcomes [19].

In this paper, we reported, for the first time, a BG predictive monitoring system based on time-series fingerstick BG readings. The results show that the stacked LSTM model can predict before- and after-meal BG in the next 7–14 days with an estimate of a 10–15% error range (MAE in proportion to mean measurements). Second, the accuracy of our model is on par with the benchmark performance of 1 h prediction models using the CGM data for type 1 and 2 diabetes [14,15,16]. As over half of the women with GDM manage their glucose with diet and exercise changes alone, the high additional cost of CGM is difficult to justify. Our results suggested that adapting the predictive glucose monitoring algorithm to routine fingerstick blood glucose monitoring has the potential to perform comparable patient monitoring as CGM and can potentially improve clinical outcomes and improve the equality of clinical resources.

Clinical machine learning models can identify patterns (digital markers) and disease phenotypes. Several machine learning approaches, including linear regression, decision trees, random forests and artificial neural networks [14,15,16,20], have been used for predicting BG continuously (every 15, 30, 45 and 60 min) with CGM glucose machines for type 1 and type 2 diabetes. In these studies, LSTM-based models outperformed other machine learning models. In our study, we used stacked LSTM as a benchmark deep learning model with fingerstick BG readings to predictively monitor the BG trend for women with GDM. LSTM networks have several advantages for BG prediction, including the ability to capture patterns in time-series data, robustness to missing data, high performance and the ability to model multivariate data. These advantages make LSTM a promising approach for BG prediction and for improving the management of diabetes and preventing of complications.

Our models predict the 1 h before- or after-meal BG for the next 7 and 14 days after each blood test, which is better scheduling of predictive monitoring for clinical intervention. In contrast, studies with CGM use a dynamic one-hour prediction window running as a background, which is beneficial for type 1 diabetes in pregnancy [21]. There is no evidence that CGM improves clinical outcomes in women with GDM. Our previous model [8] predicted which patients were likely to have glucose readings above target from those who would not in the next 72 h. However, given that the effects of medication or dietary changes may take some days, a 7- to 14-day period is more appropriate. Therefore, in women with GDM, we argue that a 7- to 14-day prediction window may be more suitable for clinical management.

One limitation of our work is that the predictive models were built using data from a single site. In addition, the fingerstick test is uncomfortable and inconvenient, especially for pregnant women. In self-monitoring settings, fingerstick blood glucose test remains the most common method due to its accuracy, low cost and portability. Although the fingerstick tests are more affordable than CGM tests, the cost of the test strips remains a significant concern in low- and medium-income counties or health systems where women or the national health system pay the direct test expense.

Another limitation is that this model did not use patient health record data. This is mainly due to data missingness. Based on our previous studies on the feature importance of a blood glucose alert system [8], we found that added static patient health data such as age and BMI help little in improving blood glucose prediction accuracy, especially when we are focusing on a short length of prediction windows, e.g., 7 or 14 days. 

In future work, we plan to develop a blood glucose prediction model by taking time-series medication information into model development. This will enable personalized precision medication management. Meanwhile, this prediction model can be improved by testing different optimisation methods and a larger number of hidden layers. In our model, adaptive moment estimation (Adam) was chosen for its stable performance and speed. To improve the model quality, other optimization methods, such as stochastic gradient descent (SGD) and momentum-based methods can be tested and compared with.

Whilst our work is a proof-of-principle, we see several potential benefits for clinical decision support to predict 7-to-14-day glucose in patients with GDM. For example, patients achieving their glucose targets could reduce the monitoring frequency. Conversely, predicted high BG readings could provoke early review by the diabetes team for medication review and dose titration. In resource-limited settings, glucose testing strips could be better rationalized. Women who dislike monitoring could be given more personalized testing schedules based on their prior readings encouraging compliance. Caution is also needed in those receiving insulin therapy where there is a risk of hypoglycaemia. Before clinical implementation, further work is needed to understand how clinicians would use the information from the algorithm and how they would explain this to patients and involve them in decision making about monitoring frequencies. We plan to expand the current study by using federated learning- or transfer learning-based LSTM architecture on multiple hospitals in the UK as the next step linking the algorithm with data in other hospitals.

## 4. Methods

### 4.1. Ethics

This study is a part of the ongoing clinical study “Predictive monitoring and management of pregnant women with gestational diabetes mellitus” (REC reference: 21/HRA/3733), which started in September 2021. This study used routinely collected anonymised, retrospective data. This study was approved by the ethical committee of the UK Health Research Authority and conducted in accordance with the Declaration of Helsinki. Participants gave their consent in writing. 

### 4.2. Data Preprocessing

We used four steps in preprocessing: data cleaning, missing data imputation, observation-prediction sequence preparation and finally splitting data into training, validation and testing sets (80:10:10 in proportions). The training and prediction datasets are from the same hospital but different patients. The training, prediction and validation data were pre-divided to avoid the leakage of information.

To clean the data, we first removed invalid tags, including “PRANDIAL-TAG-OTHER” and “PRANDIAL-TAG-NONE”. Then, we removed out-of-range BG readings <1.5 or >30, which are caused by sensor errors or human operation mistakes. The last step is to remove gestational GDays > 294 days (42 weeks 7 days) or <78 (12 weeks 0 days). After data cleaning, time-series BG data were restructured into multiple time-series BG windows.

A 5-day moving average method was used for missing data imputation by replacing missing values with the average of the values surrounding the missing value based on a specified window size. We hypothesize that blood glucose can be accurately estimated using the previous BG measurements, and that the seasonality of BG remains stable during the observational window and subsequent prediction window. For model testing on unseen data, we used the moving average on the observational window only. Sequence padding is used for the prediction windows by using an index mask to indicate the missing/invalid value (mask = 0) or true value (mask = 1). Compared to linear interpolation, the moving average method is more similar to a human’s physiological response system, which changes gradually over time based on previous BG values.

The next step is preparing the time-series sequence (observation and prediction windows) for model training, validation and testing. In this study, we designed two observation windows (7 and 14 days) and two prediction windows (7 and 14 days). The rationale for the design of seven- and fourteen-day prediction windows is based on the one- to two-week patient review period in clinical practice. This design could enable patients and clinicians to have an accurate estimate of BG status in the upcoming one to two weeks following each blood test. Each extracted sequence consists of n time steps (days) of BG readings as the observational window, and the model is trained to predict the BG at the next m steps (days). In this paper, we tested on the pairs of n = 7 and 14 and m = 7 and 14 days, namely, the prediction periods of 7 days for a 7- or 14-day prediction, and 14 days to make a 14-day prediction. Therefore, for each pregnancy, we used overlapping prediction sliding windows with fixed lengths of 14, 21 and 28 days for sequence extraction.

### 4.3. Stacked LSTM Prediction Model

In this paper, we used a deep learning model with a stacked LSTM architecture for BG predictive model development. We developed and tested on three models on three monitoring windows, namely, 7 days predict 7 days, 7 days predict 14 days, and 14 days predict 14 days. After choosing the optimized monitoring window, we then developed two independent stacked LSTM models to evaluate the model performance in the Hypo and Normal subgroups.

LSTM is a recurrent neural network (RNN) architecture used in deep learning to overcome the vanishing and exploding gradient problems that are often encountered when training time-series data [22]. The memory cell in an LSTM allows information to persist for a self-defined period of time compared to traditional RNNs. The input, output and forget gates control the flow of information into and out of the memory cell, allowing the network to selectively retain or discard information as needed. This makes LSTMs particularly effective for modelling long-term dependencies in sequential data.

**Figure 4:**
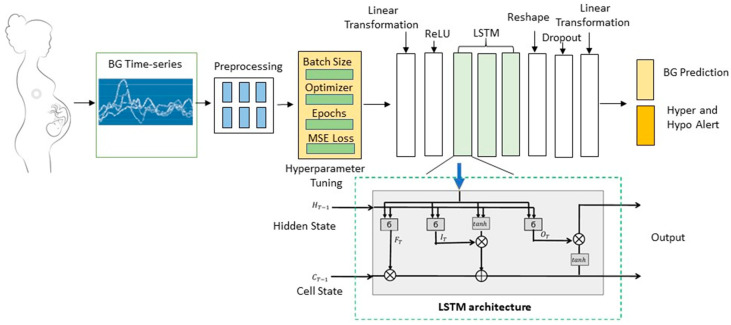
Model development pipeline and the architecture of three-layer stacked LSTM.

As shown in Figure 4, the LSTM architecture includes memory cells, gates (input, output and forget) and activation functions (Adam in our paper). The gate designs allow the LSTM to learn when to “remember” and “forget” information from previous time steps and predict sequential outputs, making it well-suited for a two-week BG prediction task. Rectified linear activation (ReLU) is an activation function used in our model. Other activation functions used within LSTM are the sigmoid for the gates and the hyperbolic tangent (tanh) function for the cell state updates and outputs, as shown in a single LSTM structure that demonstrated in the green dashed line box of Figure 4. Details of the LSTM equations are shown below. 

Let the current input be *X_T_*, and the previous hidden state is *H_T−_*_1_. Short-term state *H_T_* is the representation of BG in the most recent days, which is more likely caused by diet, exercise and medication. *C_T_* is the long-term state, and *C_T−_*_1_ is the previous long-term state. Then,

I_*T*_ = *σ*(*W*_*XI*_ ∗ *X*_*T*_ + *W*_*HI*_ ∗ *H*_*T*−1_ + *W*_*CI*_ ◦ *C*_*T*−1_ + *B*_*I*_) (1)

*F*_*T*_ = *σ*(*W*_*XF*_ ∗ *X*_*T*_ + *W*_*HF*_ ∗ *H*_*T*−1_ + *W*_*CF*_ ◦ *C*_*T*−1_ + *B*_*F*_) (2)

*O*_*T*_ = *σ*(*W*_*XO*_ ∗ *X*_*T*_ + *W*_*HO*_ ∗ *H*_*T*−1_ + *W*_*CO*_ ◦ *C*_*T*_ + *B*_*O*_) (3)

*C*_*T*_ = *F*_*T*_ ◦ *C*_*T* −1_ + *I*_*T*_ ◦ *tanh*(*W*_*XC*_ ∗ *X*_*T*_ + *W*_*HC*_ ∗ *H*_*T*−1_ + *B*_*C*_) (4)

*H*_*T*_ = *O*_*T*_ ◦ *tanh*(*C*_*T*_) (5)

Here, ∗ is the convolution operator, and *◦* is the Hadamard product (also called the elementwise product). *W_XI_*, *W_XF_*, *W_XO_*, *W_XC_*, *W_HI_*, *W_HF_*, *W_HO_* and *W_HC_* represent the convolutional filters. *B_I_*, *B_F_*, *B_O_* and *B_C_* are the biases for each layer. The input gate *I_T_* controls which part of the new input information will be kept in the long-term state. The forget gate *F_T_* decides which part of the long-term state is removed. The output gate *O_T_* decides which part of the long-term state is read. 

These equations define the forwards pass of a single LSTM cell at each time step. In a three-layer LSTM network, the hidden state from one LSTM layer is passed as input to the next two layers at each time step. The final hidden state can then be used for prediction, such as regression in our model.

Our models have three stacked LSTMs to increase the likelihood of detecting long-term dependencies of the BG in the next few days. The advantage of using a stacked LSTM network over a single-layer LSTM network is that a stacked LSTM network has a greater capacity to model complex relationships in the input data. By stacking multiple LSTM layers, the network can learn increasingly abstract representations of the input data, improving model’s ability in time-series predictions and generalising to new data [23].

We ran the LSTM architecture with a number of hyperparameter settings. The number of input channels matches the observation window (7 or 14, respectively). There is one hidden channel in each LSTM layer, totalling three hierarchal LSTM vector layers. We set the dropout to 0.2, which means the dropout probability on the outputs of each LSTM layer except the last layer. The batch size is 32, and the learning rate is 0.002. These hypermeters were set to balance execution time and performance. The final output of the three LSTM layers provides two outputs; BG prediction and hypoglycaemia alerts. The output of the BG prediction is a BG sequence responding to a fixed prediction window (7 or 14 days). In our models, we have two prediction windows, 7 or 14 days after the last BG test.

### 4.4. Model Training, Validation and Testing

The LSTM model is trained and validated under the pipeline shown in Figure 2. The inputs of the model are six time-series BG data in response to the readings before and after each meal. The readings among different tags (AB, AL, AD, BB, BL and BD) are treated as independent of each other.

As shown in Figure 2, the model training includes hyperparameter tuning via batch learning and local optimization. Batch learning is used in our model to efficiently train deep learning models. To implement batch learning in the stacked LSTM model, we divided the training data into batches of size 32. Batch normalization was carried out to regularize the activations in the network, reducing overfitting and improving generalization.

The LSTM network weights were first initialized. Within each batch, the training model performed a forward pass through the three-layer LSTM network to calculate the predictions. The mean squared error, which is the square of RMSE, is used as the loss function. The loss between the predictions of the BG sequence and the imputed BG values (preprocessed data with imputation) was calculated before performing a backward pass to calculate the gradients of the loss for weight update. After the third layer of the LSTM, Adam, an optimization algorithm, is used to perform 100-epoch hyperparameter tuning. In our experiment, the loss converges at approximately 20–30 epochs in all stacked LSTM model training. The weight of the stacked LSTM is then updated. Using batch learning can help to regularize the activations in the network, reducing overfitting and improving generalization. After the stacked LSTM models have been trained, the next step is to evaluate the models using performance metrics with the evaluation data.

At the model testing step, we used trained models to make predictions on a separate independent holdout dataset, which is 10% of the BG time series. Model testing evaluates the model generalization on unseen data and highlights issues such as model overfitting. We run each model 5 times, calculate the mean and the standard deviation of the performance metrics on the test dataset and report the mean and standard deviation of RMSE and MAE. One difference between the model test and evaluation is that we calculate the errors between the predicted values and the true values (raw data without imputation).

The performance metrics for the training, validation and test sets are the same, including RMSE and MAE. RMSE is the square root of the average squared errors before errors are averaged. MAE is the mean absolute error. The RMSE highlights the occurrence of large errors in comparison to MAE.

(6) $$\mathrm{RMSE}=\sqrt{\frac{1}{N}\sum_{i}^{N}{\left({pred}_{i}-{target}_{i}\right)}^{2}}$$

(7) $$\mathrm{MAE}=\frac{1}{N}\sum_{i}^{N}\left|\left({pred}_{i}-{target}_{i}\right)\right|$$

## Figures and Tables

**Table 1:** Stacked LSTM model performance in subgroups.

	Groups	Time Series *	RMSE (SD)	MAE (SD)
**AB: after breakfast**	Hyper (High)	20579	1.169 (0.008)	0.869 (0.005)
	Normal	21230	0.772 (0.003)	0.601 (0.004)
	Overall **	41842	**0.958 (0.007)**	0.728 (0.007)
**AL: after lunch**	Hyper (High)	18447	0.876 (0.002)	0.657 (0.003)
	Normal	21273	0.776 (0.001)	0.597 (0.001)
	Overall **	39736	**0.876 (0.003)**	0.656 (0.003)
**AD: after dinner**	Hyper (High)	14931	1.010 (0.002)	0.809 (0.002)
	Normal	22905	0.834 (0.004)	0.627 (0.005)
	Overall **	37876	**0.898 (0.003)**	0.692 (0.002)
**BB: before breakfast**	Hyper (High)	32070	0.832 (0.004)	0.472 (0.003)
	Normal	16044	0.316 (0.003)	0.242 (0.003)
	Overall **	48188	**0.622 (0.003)**	0.358 (0.004)
**BL: before lunch**	Hyper (High)	11145	1.002 (0.057)	0.720 (0.051)
	Normal	2720	0.502 (0.010)	0.419 (0.015)
	Overall **	13887	**0.814 (0.009)**	0.597 (0.010)
**BD: before dinner**	Hyper (High)	9750	0.989 (0.010)	0.655 (0.004)
	Normal	3819	0.506 (0.020)	0.400 (0.018)
	Overall **	13645	**0.845 (0.005)**	0.570 (0.004)

* Total number of BG time-series windows used in model development, evaluation and testing, split to 80:10:10 in proportions. ** The Overall group include all three subgroups: High, Normal and Hypo.

**Table 2:** Performance comparisons of GDm-Health and CGM-based LSTM models.

Study	Rabby et al. [14]	Wang et al. [15]	Doorn et al. [16]	Pustozerov et al. [17]	This Paper
**Monitoring Method**	CGM	CGM	CGM	CGM	Fingerstick
**Patient size for modelling/Diabetes Type**	6/T1DM	56/T1DM	540/mixed of normal glucose metabolism, prediabetes and T2DM	62/mixed of 48 GDM and 14 normal glucose tolerance	943/GDM
**Patient size for testing/Diabetes Type**	No independent testing	No independent testing	6/T1DM	No independent testing	105/GDM
**Prediction Model**	Three-layer stacked LSTM	(1) LSTM (2) VMD-LSTM (3) PSO-LSTM (4) VME-PSO-LSTM	LSTM	Linear regression with Lasso regularization	Three-layer Stacked LSTM
**Observation Window**	Up to 8 weeks	125 h	≥48 h	7 days	7 days
**Prediction Window**	Dynamic one-hour	Dynamic one-hour	Dynamic one-hour	One hour after meal: next day	One hour before or after meals **
Accuracy * (RMSE in mmol/L)	0.958	(1) 1.182 (2) 0.507 (3) 0.385 (4) 0.246	1.730	0.870	(1) After meal: **0.911** (2) Before meal: **0.760**

* Accuracy reported on the test set, unless there are no test set results. ** After meal is the mean of AB, AL and AD, and before meal is the mean of BB, BL and BD; results reported are the overall cohort model performance for 7-day-predicted 14-day windows.

## Data Availability

The dataset used in this study is protected under the ethical approval protocol. Code is available from the corresponding author upon request. The data preprocessing was carried out using MATLAB R2022a. Deep learning model training, testing and evaluation were performed under Python 3.8.
